# Emergence of begomoviruses and DNA satellites associated with weeds and intercrops: a potential threat to sustainable production of cassava in Côte d’Ivoire

**DOI:** 10.3389/fpls.2025.1448189

**Published:** 2025-02-26

**Authors:** Aya Ange Naté Yoboué, Bekanvié S. M. Kouakou, Justin S. Pita, Boni N’Zué, William J.-L. Amoakon, Kan Modeste Kouassi, Linda Patricia L. Vanié-Léabo, Nazaire K. Kouassi, Fatogoma Sorho, Michel Zouzou

**Affiliations:** ^1^ UPR de Physiologie et Pathologie Végétales, Laboratoire de Biotechnologie, Agriculture et Valorisation des Ressources Biologiques, UFR Biosciences, Université Félix Houphouët-Boigny (UFHB), Abidjan, Côte d’Ivoire; ^2^ The Regional Center of Excellence, Central and West African Virus Epidemiology (WAVE) for Transboundary Plant Pathogens, Pôle Scientifique et d’Innovation, Bingerville, Université Félix Houphouët-Boigny (UFHB), Abidjan, Côte d’Ivoire; ^3^ Centre National de Recherche Agronomique (CNRA), Bouaké, Côte d’Ivoire; ^4^ UFR Sciences de la Nature, Université Nangui Abrogoua (UNA), Abidjan, Côte d’Ivoire

**Keywords:** weeds, alternative hosts, cassava mosaic begomoviruses, West African Asystasia begomovirus (WAAV), alphasatellites

## Abstract

Cassava (*Manihot esculenta* Crantz) plays a significant role in the livelihoods of people in Africa, particularly in Côte d’Ivoire. However, its production is threatened by begomoviruses which cause huge yield losses. Some weeds and food crops intercropped with cassava act as reservoirs, thereby facilitating the sustenance and propagation of Cassava mosaic begomoviruses (CMBs), along with other begomoviruses. To effectively manage these diseases, it is imperative to enhance our understanding of the various hosts of cassava viruses in Côte d’Ivoire. Thus, a comprehensive nationwide survey was conducted in 2017 in cassava fields across Côte d’Ivoire, and molecular analyses were performed on the samples collected. The results obtained from this survey indicated that 65 plant species belonging to 31 families were potential alternative hosts for CMBs in Côte d’Ivoire. The molecular analyses revealed that four species, *Capsicum annuum*, *Solanum melongena*, *Centrosema pubescens*, and *Asystasia gangetica* exhibited differential affinities for both African cassava mosaic virus and East African cassava mosaic Cameroon virus. Additionally, other begomoviruses and new alphasatellites were identified. Soybean chlorotic blotch virus was isolated from *C. pubescens* while West African Asystasia virus 1, West African Asystasia virus 2, and a new Asystasia yellow mosaic alphasatellite were isolated from *A. gangetica* which appears to be a plant species that could favor the emergence of new viral species harmful to cassava cultivation. This study offers insights that will inform the development of more effective control methods for sustainable cassava production in Côte d’Ivoire.

## 1 Introduction

Begomoviruses are a major impediment to the production of several domesticated food and industrial crops, resulting in significant yield losses. Cassava (*Manihot esculenta* Crantz), an important and strategic crop in Côte d’Ivoire (FAO, 2021), is threatened by many viral pathogens, particularly begomoviruses. The economic repercussions of these infections on cassava are substantial, amounting to billions of US dollars annually on a global scale (Leke et al., 2015). Begomovirus is a genus of emerging viruses belonging to the family Geminiviridae. The emergence of these plant viruses can be attributed not only to an increase in vector populations, the exchange of crop germplasm, changes in cropping systems (e.g., extensive agriculture), and host range expansion (Lefeuvre et al., 2007). Furthermore, the evolution of begomoviruses and their emergence can be influenced by the existence of other viruses, various hosts, and changing environmental conditions (Jones, 2014).

Begomoviruses belong to the genus *Phytovirus*, according to the International Committee on Taxonomy of Virus (Fiallo-Olivé et al., 2021). Contrary to other genera, begomovirus has monopartite or bipartite genomes (Zerbini et al., 2017). The genomes of bipartite begomoviruses consist of two components: DNA-A and DNA-B, each of 2.8 kb. The DNA-A component encodes all virus functions required for DNA replication, gene expression, and insect transmission, while DNA-B is responsible for systemic infection. The DNA-A component of bipartite begomoviruses encodes six open reading frames (ORFs), including the coat protein, the replication-associated protein or AC1 protein, the transcriptional activator protein (TrAP) or AC2 protein, the replication enhancer protein (REn) or AC3 protein, the symptom determinant protein or AC4 protein. However, the presence of AC5/C5 ORF has also been reported in certain bipartite begomoviruses (Fontenelle et al., 2007 **;** Kheyr-Pour et al., 2000). Viral ORFs are separated by an intergenic region (IR) possessing a common area (CR) that consists of conserved nucleotides between DNA-A and DNA-B (Leke et al., 2015). The common region possesses the origin of replication (ori), stem-loop-like non-nucleotide sequence (TAATATT↓AC), and two bidirectional RNA polymerase II promoters (Fiallo-Olivé and Navas-Castillo, 2020). DNA-B possesses two ORFs: BV1 in a sense and BC1 in an antisense orientation (Krenz et al., 2012 *)*.

Begomoviruses are transmitted by the whitefly *Bemisia tabaci* in a (semi-)persistent circulative manner within a few hours of virus acquisition (Seal et al., 2006). *B.* tabaci is the only known vector species for begomoviruses, and hence, the global distribution of begomoviruses is closely related to that of this species. In West Africa, several begomoviruses, such as ACMV, EACMCMV, and EACMV caused damage in cassava fields. Recently, in Guinea, East African cassava mosaic virus Uganda (EACMV-Ug) was found in all over the country for the first time (Combala et al., 2024). This begomovirus devasted cassava plantations in Uganda during the 1990s epidemic. Also, in recent years, food crops such as tomato, okra, and pepper intercropped in cassava fields were found infected by begomoviruses in many countries in Africa (Benin, Burkina Faso, Cameroon, Ghana, Côte d’Ivoire, Mali, Niger, Nigeria, Senegal, and Togo) (Tiendrébéogo et al., 2010 **;** Leke et al., 2015 **;** Assiri et al., 2017 **;** Mivedor et al., 2017 **;** Ouattara et al., 2020). Moreover, scientific reports have documented that weeds also serve as reservoirs or alternative hosts for the maintenance and spread of begomoviruses (Alabi et al., 2008 **;** Monde et al., 2010 **;** Prajapat et al., 2013 **;** Leke et al., 2015 **;** Fiallo-Olivé et al., 2017 **;** Eni et al., 2021). This is the case of *Senna occidentalis* (L.) Link, *C. pubescens* Benth., *Combretum confertum* (Benth.) Michael Adam Lawson, *Pueraria javanica* (Benth.) Benth., *Leucaena leucocephala* (Lam.) de Wit, *Glycine max* L. in which different cassava mosaic begomoviruses (CMBs) and non-cassava mosaic begomoviruses (non-CMBs) were found. Some begomovirus species require DNA satellites to induce symptoms in their host (Briddon and Stanley, 2006). DNA Satellites depend on helper viruses for replication, encapsidation, and transmission, are unique to plant viruses, and are associated with diseases. Of the three groups of satellites, betasatellites play a significant role in increasing the virulence of begomoviruses (Kumar et al., 2018). Generally, alphasatellites are shown to attenuate disease symptoms and reduce virus accumulation (Mar et al., 2017). Some betasatellites have acquired the ability to transreplicate with noncognate begomoviruses, but replication is supported by their cognate helper viruses better than non-cognate helper viruses (Xu et al., 2019). Moreover, the identification of alphasatellites in weeds has been reported in West and Central Africa by Leke et al. (2015). De Bruyn et al. (2015) discovered in Madagascar that *A. gangetica L.* was infected by Asystasia mosaic Madagascar virus (AMMGV), a bipartite begomovirus (De Bruyn et al., 2015) similar to West African Asystasia virus 1 (WAAV1) reported to infect not only *A. gangetica* but also cassava in Cameroon and Togo (Leke et al., 2016). *A. gangetica* can adapt easily to different ecological conditions and has thus colonized several cultivated areas. Its presence in cassava fields poses a real threat. In Côte d’Ivoire, *A. gangetica* is mainly found as a weed in the vicinity and within cassava fields and usually displays pronounced mosaic symptoms characteristic of virus infections.

Better knowledge of the alternative hosts of begomoviruses in Côte d’Ivoire will enable a better understanding of the epidemiology of Cassava Mosaic Disease (CMD). Therefore, the objective of this study is to assess the dynamics of viral species exchange between weeds, associated crops, and cassava to develop appropriate control measures to alleviate the burden of the CMD on cassava in Côte d’Ivoire.

## 2 Material and methods

### 2.1 Survey and sampling

**Figure 1:**
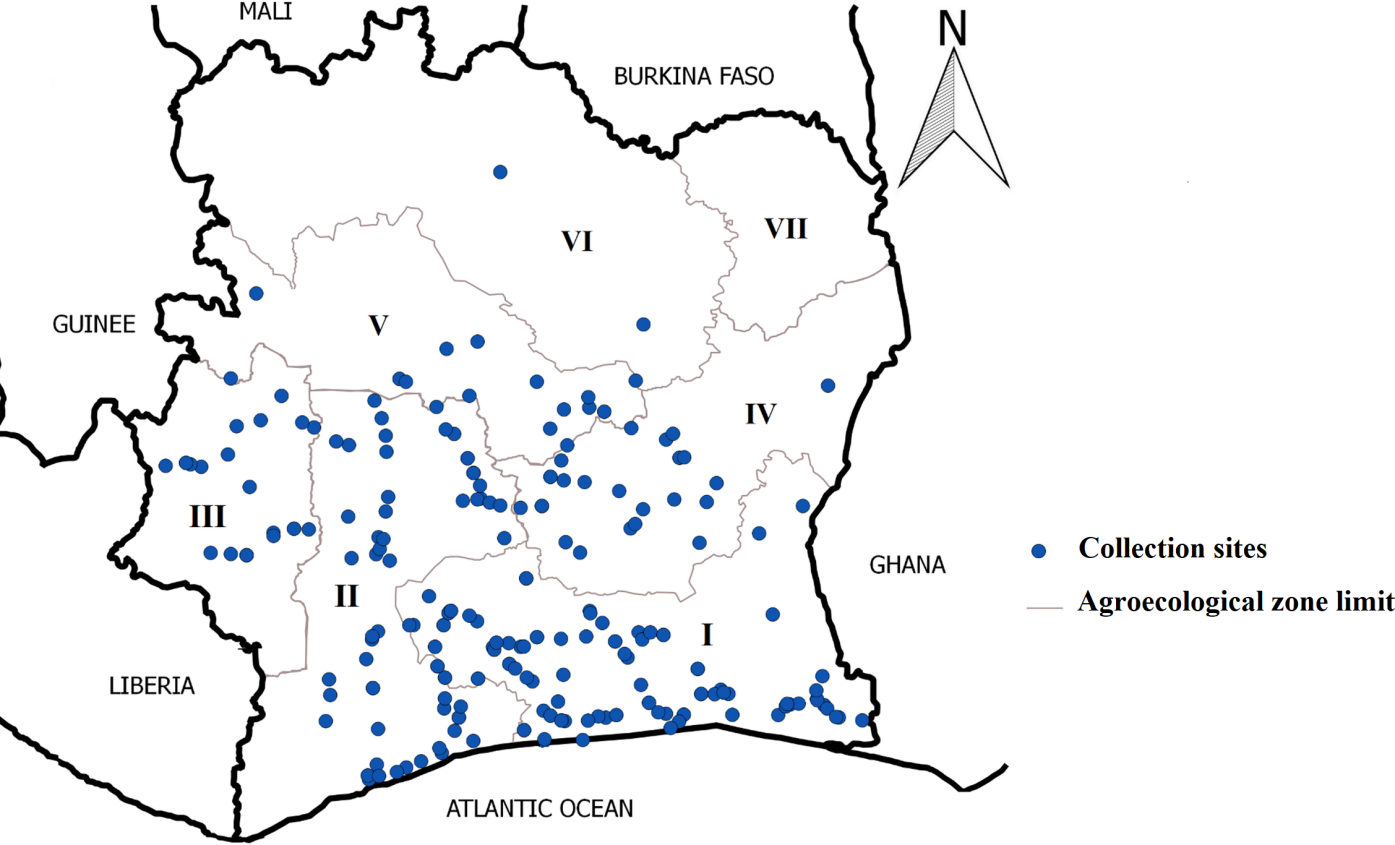
Map of Côte d’Ivoire showing the different areas surveyed and sample collection sites.

In September 2017, cassava fields were surveyed in six agroecological zones in Côte d’Ivoire (**Figure 1**). Surveys were conducted along the main roads and cassava fields located 8 to 10 km apart were assessed following a modified protocol described by Sseruwagi et al. (2004). During these surveys, weeds and other intercropped plants in cassava fields were assessed for the presence or absence of disease symptoms (mosaic, distortion, curling, filiform, stunted growth). Furthermore, the Centre National de Recherche Agronomique (CNRA), cassava conservation plots in Bouaké and Man were also surveyed. A total of 306 leaf samples were collected from non-cassava plants. These samples were placed in envelopes and stored at room temperature in the laboratory before molecular analysis.

### 2.2 Detection of CMBs in weeds and intercrops with specific primers

Total DNA extraction from leaf samples from weeds and other intercrops was performed using the CTAB protocol as previously described by Doyle and Doyle (1987). The concentration of extracted total DNA was set at 50 ng/µl with a spectrophotometer (Eppendorf) and 5 µl was used for PCR. Detection of African Cassava Mosaic Virus (ACMV), East African Cassava Virus (EACMV), and East African Cassava Cameroon Virus (EACMCMV) were performed using several specific primers (**Table 1**). The Polymerase Chain Reaction (PCR) mixture consisted of 0.625 U GoTaq polymerase (Promega), 1 X GoTaq Reaction Buffer (Promega), 0.2 mM of dNTP (NEB), 1 mM MgCl2 (Promega), and 0.4 µM of each primer (Eurogentec). The PCR program consisted of an initial denaturation step at 94°C for 4 minutes, followed 35 cycles of 94°C for 1 minute, 55°C for 1 minute, and 72°C for 1 minute, and a final extension of 72°C for 10 minutes. We performed electrophoresis of the PCR products on a 1% agarose gel stained with ethidium bromide and visualized under UV light. Furthermore, the PCR products were sequenced by the Sanger method at GENEWIZ (Germany).

**Table 1:** Primer pairs used for the detection and/or amplification of the full genomes of ACMV, EACMV, and EACMCMV.

Primer	Primer sequence	Virus/DNA target	Size/Amplified region
ACMVB1	5’-TCG GGA GTG ATA CAT GCG AAG GC-3’	ACMV-DNA-B	628 pb/BV1-BC1
ACMVB2	5’-GGC TAC ACC AGC TAC CTG AAG CT-3’		
ACMV21F	5’-GCA GTG ATG AGT TCC CCG GTG CG-3’	ACMV, EACMV, EACMCMV, EACMKV, EACMCMV EACMZV, SACMV/DNA-A	552 pb/AC3-AC2-AC1
ACMV21R	5’-ATT CCG CTGC GCG GCC ATG GAG ACC-3’		
CMBRepF	5’-CRT CAA TGA CGT TGT ACC A-3’	EACMV/DNA-A	650 bp/AC1
EACMVRepR	5’-GGT TTG CAG AGA ACT ACA TC-3’		
VNF031	5’-GGA TAC AGA TAG GGT TCC CAC -3’	EACMCMV/DNA-A	560 pb/AC2-AC3
VNF032	5’-GAC GAG GAC AAG AAT TCC AAT-3’		
VNF007	5’-GGC-CTA-GGG-GCT-GTG-AAG-GYC-CCA-3’	ACMV/DNA-A	2800 pb
VNF008	5’-GGC-CTA-GGT-ATG-TCT-GGG-CTT-C-3’		
VNF021	5’-CTC-ATC-GAT-ATA-GGG-TAT–TGC-3’	ACMV/DNA-B	2800 pb
VNF022	5’-CTC-ATC-GAT-GCT-GTT-GAT-TAT-3’		
VNF003	5’-CCC-AAG-CTT-TGG-TTA-GAG-GTT-T-3’	EACMCMV/DNA-A	2800 pb
VNF004	5’-CCC-AAG-CTT-GTT-CCT-TCA-TCC-CWA-3’		
EB03	5’-GTT-AAC-ATT-TAT-TTT-TTG-TTT-MTC-GC-3’	EACMCMV/DNA-B	2800 pb
EB04	5’-GTT-AAC-GAA-ATA-AAA-GYW-GAA-CGT-3’		

### 2.3 Molecular characterization of full genomes of CMBs and non-CMBs from weeds and intercrops

To generate a full-length genome of cassava mosaic begomoviruses (CMBs), selected DNA samples were amplified using full-length genome primers (**Table 1**). The PCR reactions were carried out using Pfu polymerase (Promega), according to the manufacturer’s protocol. The PCR amplification profile consisted of an initial denaturation step at 94°C for 4 minutes, followed by 35 cycles of 94°C for 1 minute, 58°C for 1 minute, and 72°C for 3 minutes, and a final extension of 72°C for 15 minutes. The full genome obtained was cloned into a pGEMT-Easy vector (Promega).

For the characterization of non-CMBs, DNA samples were amplified by rolling circle amplification (RCA) using the TempliPhi 100 kit (GE Healthcare) (Inoue-Nagata et al., 2004). The RCA products were digested with one of the endonucleases *Bam*HI, *HindIII*, *NdeI*, *EcoRI* or *PstI* assembled to yield the full-length genomes (~2.8 kb). The DNA fragments were cloned into the vector pGEM-3Zf (+) (Promega Corp., Madison, WI, USA). The cloned products were sequenced by the primer walking method by GENEWIZ (Germany).

### 2.4 Phylogenetic analysis

All the sequences were analyzed with Geneious Prime version 2022.2.1. The sequences were trimmed and assembled *de novo*. Consensus sequences obtained were subjected to a BLASTn search and aligned separately with begomovirus isolates’ nucleotide sequences from GenBank, using the MEGAX clustalW function (Kumar et al., 2018). Maximum likelihood trees were constructed in MEGAX using T92+G and GTR+G nucleotide substitution models according to MEGAX and a bootstrap of 1000. Model fitting was performed using MEGA-X. Thus, the model with the lowest Bayesian Information Criterion (BIC) was considered the one with the best model description. Phylogenetic trees were visualized and edited using FigTree v1.4.3 (Edinburgh, UK). Then, Pairwise Sequence Comparison identity was performed using SDT v. 1.2 with pairwise deletion of gaps (Muhire et al., 2014).

## 3 Results

### 3.1 Potential alternative hosts of CMBs

During the surveys, 65 plant species belonging to 31 families showed typical CMD symptoms (mosaic, deformation, and filiform) (**Table 2**). These included weeds and intercrops. Among these plant species, *A. gangetica* and *C. pubescens* were the most preponderant in the cassava fields surveyed. They were mainly found in agroecological zones I, II, and III (**Table 2**).

**Table 2:** List of potential host plant species found in cassava fields based on CMD symptoms.

Family	Species	Number of samples	Symptoms	Potential host type	AEZ
Acanthaceae	*Asystasia gangetica* (L.) T	87	M, LD	Weed	I, II, III, IV, V
	*Justica flava* (Forssk.)	1	M	Weed	III
Amaranthaceae	*Amaranthus* sp*inosus* L.	1	M	Weed	IV
Anacardiaceae	*Rhus longipes* Engl.	1	M	Weed	III
Apocynaceae	*Funtumia africana* (Benth.) Stapf	1	M	Weed	II
	*Holarrhena floribunda* (G. Don) Dur. & Schinz	1	M	Weed	II
	*Parquetina nigrescens* (Afzel.) Bullock	1	M	Weed	I
	*Rauvolfia vomitoria* (Afzel)	14	M	Weed	I, II, III, IV, V
Araceae	*Xanthosema mafaffa* Schott	2	M	Intercrop	I, IV
Asteraceae	*Ageratum conyzoïdes* L.	1	M	Weed	I
	*Chromolaena odorata* L.	2	M	Weed	I; II
	*Synederla nodiflora* (L.) Guertn.	4	M	Weed	I
	*Vernonia amygdalina* Delile	1	M	Weed	II
Bignoniaceae	*Spathodea campanulata* Beauv.	1	M	Weed	II
Bombacaceae	*Bombax buonopozense* P. Beauv.	2	M	weed	I, II
Caesalpiniaceae	*Cassia hirsuta* (L.) H.S. Irwin & Barneby	8	M	Weed	I, II, III, IV, V, VI
	*Cassia occidentalis* L.	1	M	Weed	VI
Caricaceae	*Carica papaya* L.	2	M	Intercrop	I, IV
Convolvulaceae	*Ipomea obcura* (L.) Ker Gawl.	1	M	Intercrop	I
Cucurbitaceae	*Cucurbita pepo* L.	1	M	Intercrop	IV
	*Momordia charantia* L.	2	M	Weed	I
Dioscoreaceae	*Dioscorea cayenensis* Lam.	1	M	Intercrop	II
Euphorbiaceae	*Alchornea cordifolia* (Schumach. & Thonn.)	1	M	Weed	II
	*Euphorbia herophylla* Linn.	1	M	Weed	II
	*Hevea brasiliensis* (Willd. ex A.Juss.) Müll.Arg.	1	M	Intercrop	I
Fabaceae	*Baphia nitida* Lodd.	2	M	Weed	I, III
	*Centrosema pubescens* Benth.	57	M, LD	Weed	I, II, III, IV, V
	*Crotalaria retusa* L.	1	M	Weed	I
	*Desmodium tortuosum* (Schwartz) DC	1	M	Weed	IV
	*Millettia zechiana* Harms	1	M	Weed	II
	*Mucuna pruriens* (L.) DC.	5	M	Weed	II, III, IV
	*Phaseolus vulgaris* L. [cult.]	3	M	Intercrop	I, II
	*Pueraria phaseoloides* Roxb	3	M	Weed	I
Icacinaceae	*Icacina mannii* Oliv.	1	M	Weed	I
Lamiaceae	*Ocimum gratissimum* L.	2	M	Weed	I, III
	*Clerodendrum* sp*lendens* G. Don	9	M	Weed	I, II, III
	*Clerodendrum umbellatum* Poir.	4	M	Weed	I, II
	*Clerodendrum volubile* P. Beauv	3	M	Weed	I, II
	*Stachys sylvatica* L.	2	M	Weed	IV
Gentianaceae	*Anthocleista djalonensis* Chev.	1	M	Weed	I
Malvaceae	*Abelmoschus esculentus* L.	2	M	Weed	IV
	*Sida acuta* Burm f.	8	M	Weed	I, II, IV
	*Sida cordifolia* Linn.	1	M	Weed	IV
	*Sida rhombifolia* L.	1	M	Weed	III
	*Sida urens* Linn.	4	M	Weed	III, IV, VI
Mimosaceae	*Albizia zygia* (DC.) Macbr.	9	M	Weed	I, II, IV, V
Moraceae	*Antiaris toxicaria* Lesch.	1	M	Weed	III
	*Ficus benjamina* L.	2	M	Weed	I
	*Ficus exasperata* Vahl.	9	M	Weed	I, II, IV, V
	*Ficus sur* Forsk.	1	M	Weed	I
Passifloraceae	*Adenia guineesis* W.J. de Wilde	1	M	Weed	III
	*Passiflora foetida* L.	4	M	Intercrop	I, II, IV
Poaceae	*Sorghum halepense* L. Pers.	1	M	Intercrop	IV
	*Zea mays* L.	1	M	Intercrop	II
Rubiaceae	*Chassalia kolly* Schumach.	1	M	Weed	II
	*Psydrax parviflora* Afzel.	5	M	Weed	I, III, V
Sapindaceae	*Cardiospermum halicacabum* Linn.	1	M	Weed	I
Solanaceae	*Capsicum annuum* L.	9	M, LD	Intercrop	I, II, IV, VI
	*Solanum torvum* Sw.	1	M	Weed	I
	*Solanum melongena* L.	8	M, LD	Intercrop	I, II, V, VI
Sterculiaceae	*Sterculia tragacantha* Lindl.	1	M	Weed	II
Urticaceae	*Pouzolzia guineensis* Benth.	1	M	Weed	II
Verbenaceae	*Stachytarpheta indica* (L.) Vahl	2	M	Weed	I, II
Vitaceae	*Cissus polyantha* Glig & Brandt	1	M	Weed	I
Zingiberaceae	*Zingiber officinale* Roscoe	1	M, F	Intercrop	I

M, mosaic; LD, leaf distortion; F, filiform.

### 3.2 Weeds and intercrops infected by CMBs

**Figure 2:**
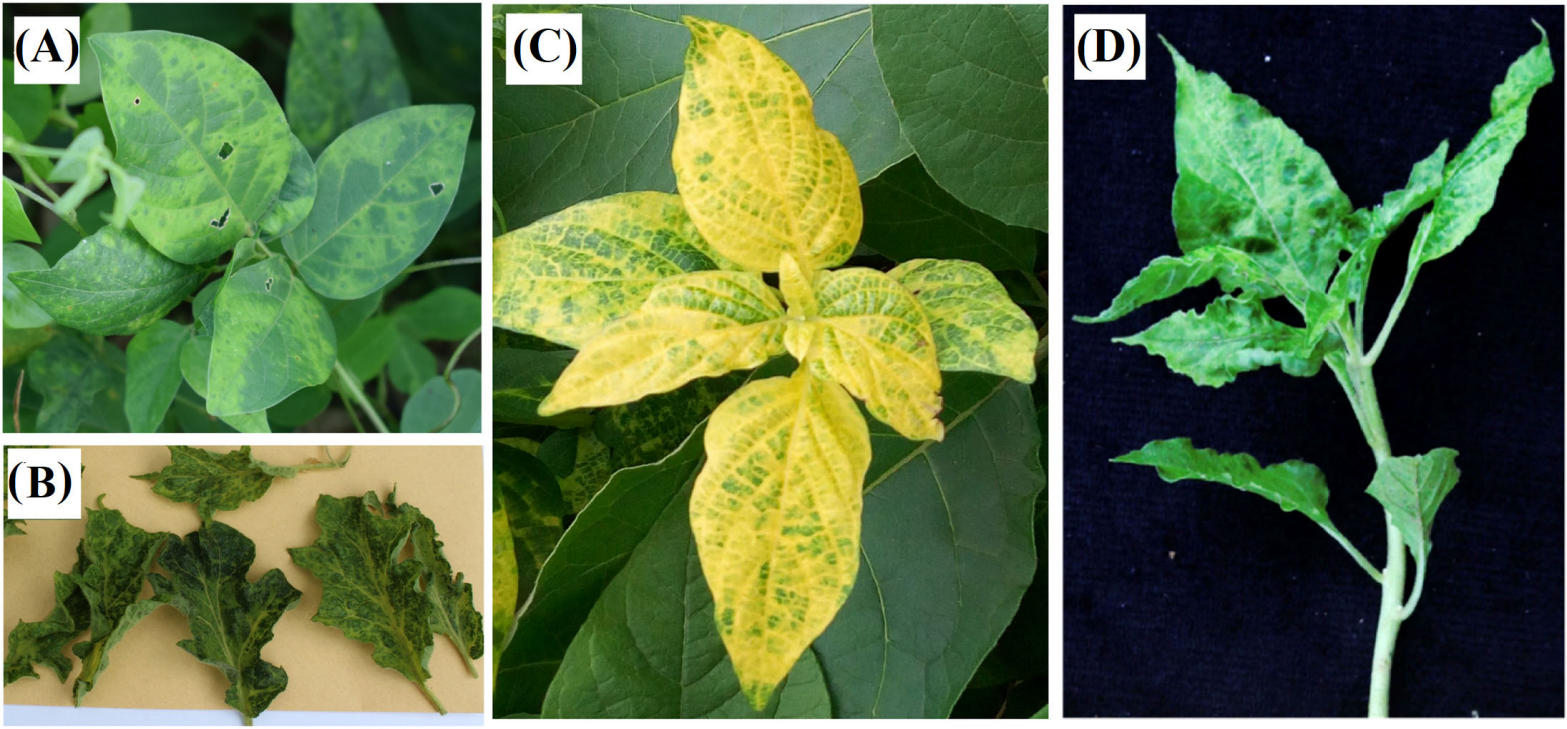
Pictures of potential alternative hosts of Cassava mosaic begomoviruses (CMBs) in Côte d’Ivoire. **(A)** leaves of *C. pubescens* showing mosaic, **(B)** leaves of *S. melongena* displaying mosaic and leaf distortion, **(C)** leaves of *A*. *gangetica* showing mosaic and leaf distortion, **(D)** leaves of *C. annuum* displaying mosaic and leaf distortion.

**Figure 3:**
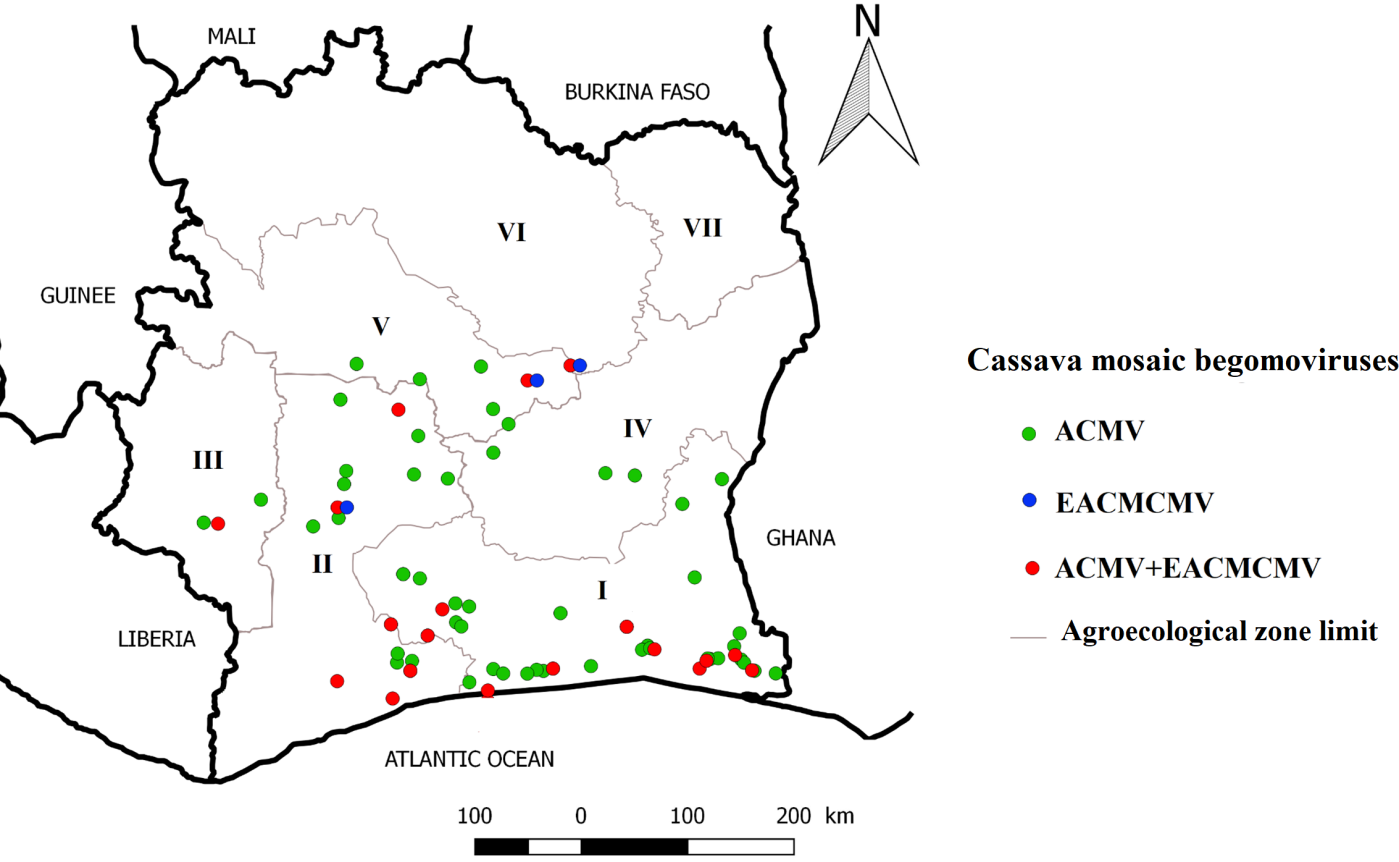
Distribution map of Cassava mosaic begomoviruses isolated in alternative hosts.

A total of 306 samples were tested with CMB-specific primers. The plants that tested positive for PCR belong to three families Acanthaceae (*A. gangetica*), Fabaceae (*C. pubescens*), Solanaceae (*C. annuum* and *S. melongena*) (**Figure 2**). An overall amplification rate of 50.98% (156/306) was obtained. Direct sequencing of PCR products identified two CMB species, ACMV and EACMCMV. The results obtained from the analysis are presented in **Table 3**. A total of seven full genome sequences of ACMV generated have been successfully deposited in the DDBJ/ENA/GenBank database (https://www.ddbj.nig.ac.jp/ddbj/submission.html) with the following accession numbers: LC723864, LC723865, LC724017, LC724016, LC723868, LC723867 and LC723866. For EACMCMV, two partial genome sequences were deposited (LC723877 and LC723878). The distribution of ACMV and EACMCMV isolated in these alternative hosts is shown in **Figure 3**.

**Table 3:** Percentage of detection of Cassava mosaic begomoviruses (CMBs) in alternative host plants by PCR and sequencing.

Family	Species	ACMV	EACMCMV	ACMV+EACMCMV
Acanthaceae	*A. gangetica* (L.) T	58.54% (48/82)	0% (0/82)	31.71% (26/82)
Fabaceae	*C. pubescens* Benth.	63.16% (36/57)	0% (0/57)	0% (0/57)
Solanaceae	*C. annuum* L.	11.11% (1/9)	33.33% (3/9)	11.11% (1/9)
	*S. melongena* L.	37.5% (3/8)	0% (0/8) ^1^	12.5% (1/8)

### 3.3 Non-CMBs obtained by PCR and RCA

PCR with CMB primers followed by sequencing revealed the presence of non-CMBs in some samples tested. The rate of amplifications of non-begomovirus was 3.92% (12/306). The primers ACMV21F/ACMV21R amplified Soybean chlorotic blotch virus (SbCBV) in *C. pubescens* and West African Asystasia virus 1 (WAAV1) in *A. gangetica* (**Table 4**). The primers CMBRepF/EACMVRepR revealed the presence of West African Asystasia virus 2 (WAAV2) and West African Asystasia virus 1 (WAAV1) in *A. gangetica*.

**Table 4:** Percentage of detection of non-Cassava mosaic begomoviruses (CMBs) and alphasatellites in alternative host plants by PCR and sequencing.

Family	Plant species	WAAV2	WAAV1	SbCBV	Alphasatellites
Acanthaceae	*A. gangetica*	3.65% (3/82)	9.75% (8/82)	0% (0/82)	1.21% (10/82)
Fabaceae	*C. pubescens*	0% (0/57)	0% (0/57)	1.75% (1/57)	0% (0/57)

The sequencing products of RCA confirmed infection of *A. gangetica* by WAAV1 and WAAV2 and revealed the presence of DNA satellites (**Table 4**). A total of three WAAV2 sequences have been deposited in the Genbank with LC723950, LC723876, and LC723951 as accession numbers. For WAAV1, seven sequences have been deposited (LC723869, LC723870, LC723871, LC723872, LC723873 LC723874 and LC723875) and for the alphasatellites, ten sequences have been deposited (LC724018, LC724019, LC724021, 022, LC724060, LC724061, LC724062, LC724063, LC724064 and LC724020).

#### 3.3.1 CMBs sequences

Cloning and sequencing efforts yielded a total of seven complete genomes of ACMV including two DNA-A and five DNA-B. Two genome sequences (2871 nt for both) of ACMV DNA-A were obtained from *C. pubescens* samples. Regarding ACMV DNA-B, three sequences (2722 nt for the three) were obtained from *C. pubesens* and two sequences (2,721 and 2719 nt) from *A. gangetica*. For partial genomes, we obtained two sequences of EACMCMV (1427 and 1578 nt) from only the *A. gangetica* sample.

The two complete sequences corresponding to the ACMV DNA-A (2781 nt) isolated from *C. pubesecens* (LC723864, LC723865), shared a nucleotide identity of 96.66% to 97.70% with isolates from cassava from West, East (Uganda) and Central Africa.

The five complete sequences of ACMV DNA-B (2719-2722 nt) isolated from *A. gangetica* (LC723867 and LC723866) and *C. pubescens* (LC724017, LC724016, and LC723868) shared 91.11% to 94.68% nucleotide identity with cassava virus isolates. The LC723868 and LC723866 sequences shared 97.39% and 97.35% identity sequence respectively with the Ghana isolate (JN165086).

The two partial sequences of EACMCMV (1427-1578 nt) isolated from *A. gangetica* (LC723878 and LC723877) shared 93.15% and 97.33% identity with the Burkina Faso isolate (LC659083). The Query cover of these 2 sequences varied from 65 to 68%. BLASTn search in GenBank database (NCBI, BLASTn) of the remaining portions indicates that they are 98% similar to the DNA-B of EACMCMV isolate Ghana (JN165087).

**Figure 4:**
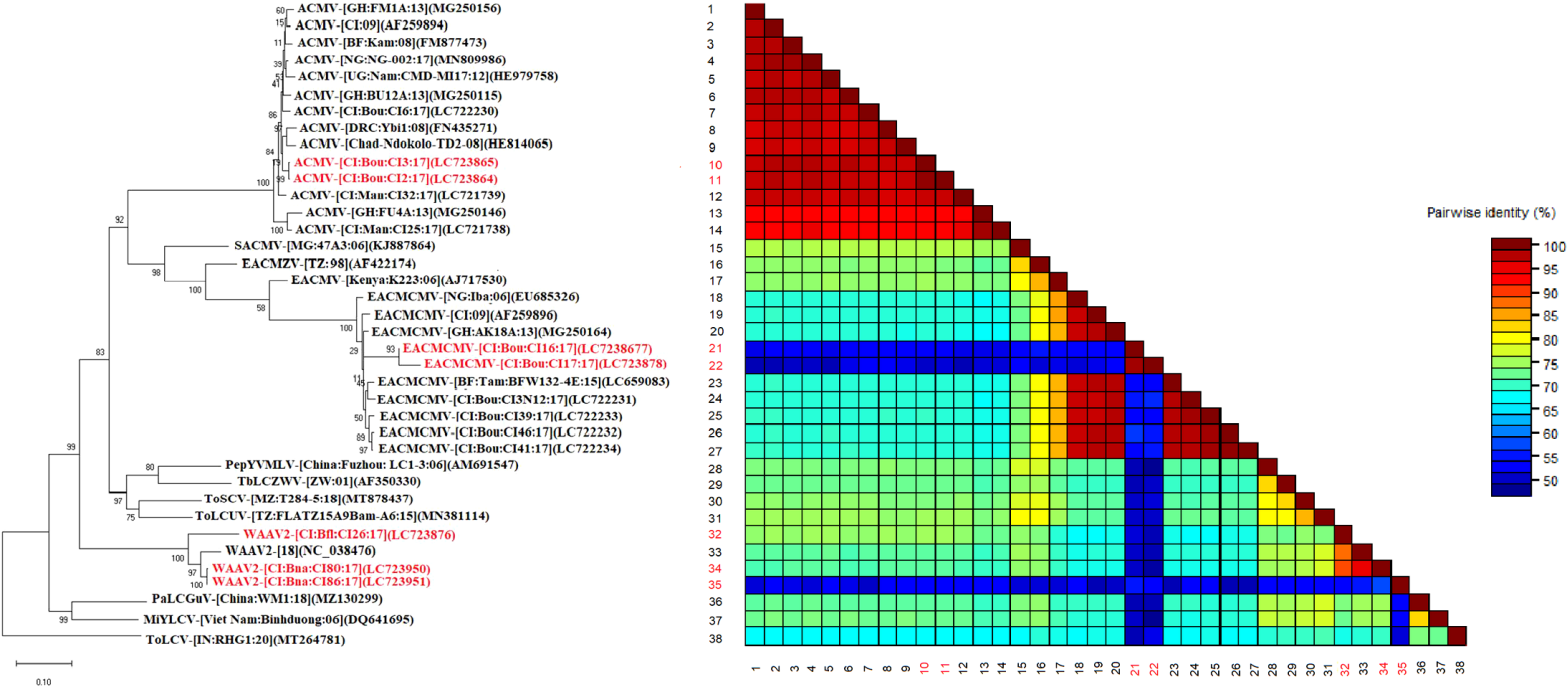
Maximum likelihood phylogenetic tree indicating the relationships between Côte d’Ivoire isolates of African cassava mosaic virus (2 isolates), East African cassava mosaic Cameroon virus (2 isolates), and West African Asystasia virus 2 (WAAV2; 3 isolates) obtained from *C. pubescens* and *A. gangetica* and diverse representative begomoviruses DNA-A isolates. The tree is based on sequences of ACMV (DNA-A), EACMCMV (DNA-A), WAAV2 (DNA-A) and rooted using Tomato leaf curl virus (GenBank accession, DNA-A: MT264781); as an outgroup. The sequences obtained in this study are in red while those in black were taken from GenBank. Bootstrap analysis was performed with 1000 replicates. The matrix uses a discontinuous range of color (red, yellow, green, and blue) to differentiate two cut-off values representing the species (< 91% yellow- green-blue), and the isolate (> 94%, red) demarcation thresholds of begomoviruses.

**Figure 5:**
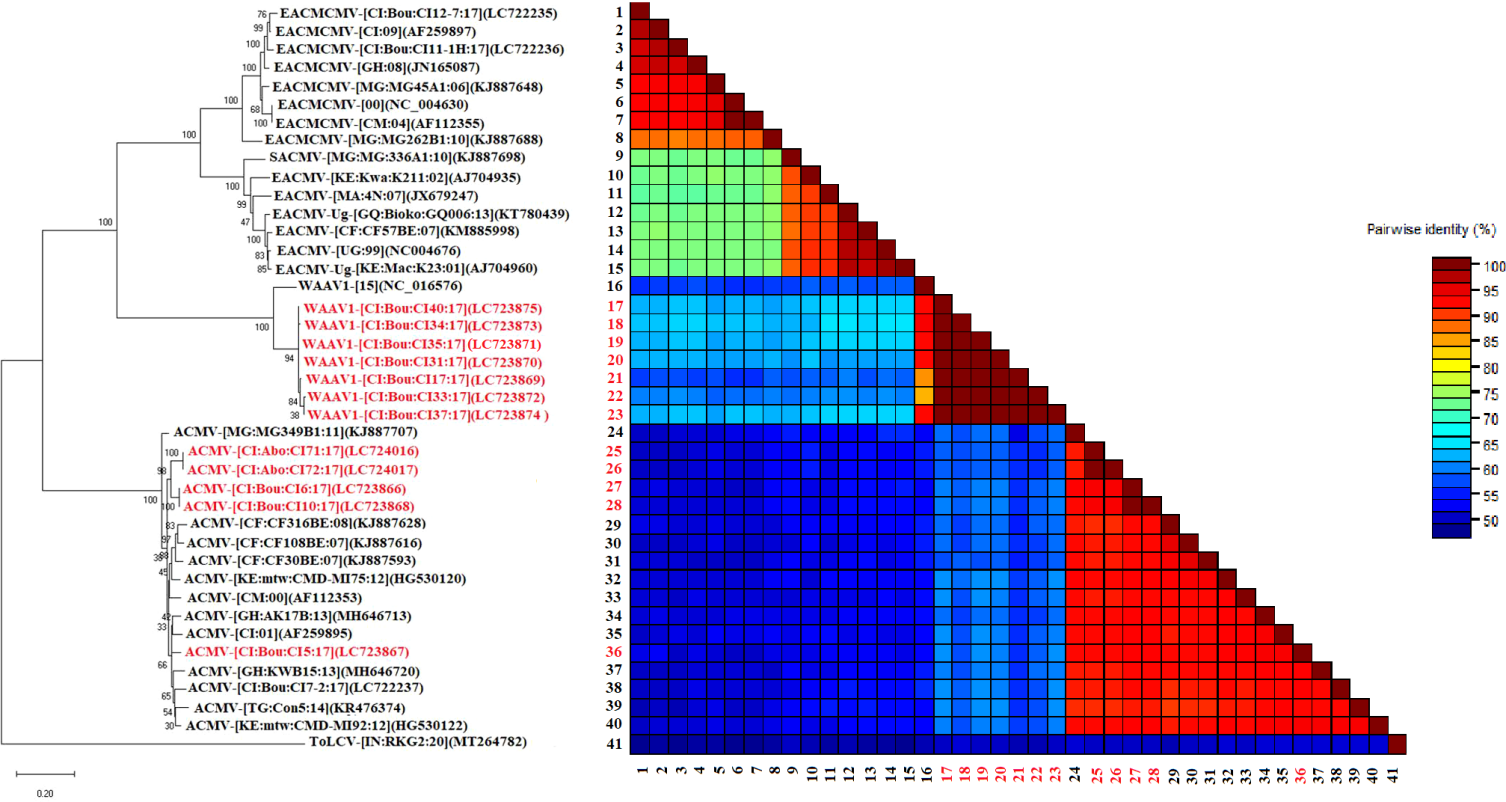
Maximum likelihood phylogenetic tree indicating the relationships between Côte d’Ivoire isolates of African cassava mosaic virus (5 isolates) and West African Asystasia virus 1 (WAAV1; 7 isolates) obtained from *C. pubescens* and *A. gangetica* and diverse representative begomoviruses DNA-B isolates. The tree is based on sequences of ACMV (DNA-B), and WAAV1 (DNA-B) and rooted using Tomato leaf curl virus (GenBank accession, DNA-B: MT264782); as an outgroup. The sequences obtained in this study are in red while those in black were taken from GenBank. Bootstrap analysis was performed with 1000 replicates. The matrix uses a discontinuous range of color (red, yellow, green, and blue) to differentiate two cut-off values representing the species (< 91% yellow- green-blue), and the isolate (> 94%, red) demarcation thresholds of begomoviruses.

Phylogenetic analyses constructed using the maximum likelihood (ML) showed that ACMV DNA-A grouped in one cluster with sequences from West, Central, and East Africa (**Figure 4**). Pairwise comparisons using the SDT program showed that the sequences obtained in this work were closely related and showed 90–100.0% similarity (**Figure 4** **).** The DNA-A sequences of EACMCMV formed a cluster with the West African isolates of EACMCMV species (Côte d’Ivoire, Burkina Faso, Ghana, and Nigeria) presented in **Figure 4**. However, the pairwise nucleotide sequence identity of EACMCMV DNA-A isolates obtained in this study was found very low (50-60%) with EACMCMV isolates in NCBI (**Figure 4** **).** The DNA-B isolates identified in this study also were grouped in a single cluster with the West, Central, and East African isolates. The SDT matrix uses the color blue to show the low similarity (50–60.0%) between sequences (**Figure 5**).

#### 3.3.2 Non-CMBs sequences

Sequence analysis of the non-CMBs revealed that the WAAV2 sequences obtained correspond to the DNA-A component, including one complete sequence and two partial sequences. The WAAV1 sequences obtained corresponded to the DNA-B component, including one complete sequence and 6 partial sequences. The complete DNA-A sequence of WAAV2 (LC723950) obtained in this study shared a 94.91% nucleotide identity with the West African isolate (NC_038476) which corresponds to a monopartite genome. The partial sequence of DNA-A of WAAV2 of approximately 2545 nt (LC723876) shared a nucleotide identity of 91.04% with the isolate NC_038476This appears to be a new strain of the WAAV2.

DNA-B of WAAV1 complete genome (2663 nt) and six partial sequences (1116-1429 nt) with accession numbers LC723869, LC723870, LC723871, LC723872, LC723873 LC723874, and LC723875 shared a nucleotide identity of 90.81% to 96.78% with isolates from West and Central Africa (Benin, Nigeria, and Cameroon).

Phylogenetic analyses constructed using the maximum likelihood (ML) showed that the DNA-A sequences of the monopartite WAAV2 form one cluster with the West African isolate NC_038476. Pairwise comparisons using the SDT program showed that the sequences obtained in this work were closely related and showed 90–100.0% similarity (**Figure 4**). In addition, the DNA-B sequences of the bipartite virus WAAV1, form one cluster with the West African isolates (**Figure 5**).

**Figure 6:**
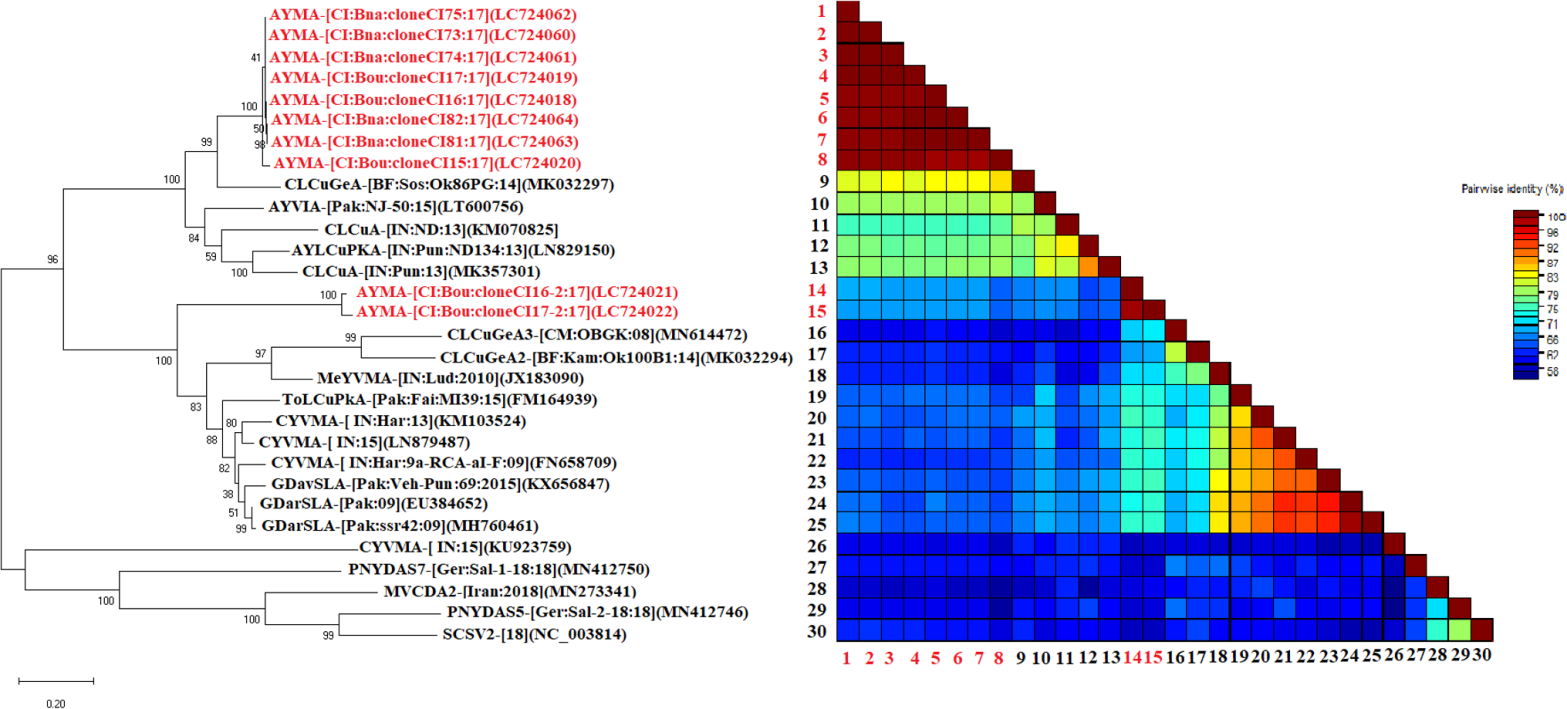
Maximum likelihood phylogenetic tree indicating the relationships between Côte d’Ivoire isolates from *A. gangetica* of alphasatellites (10 isolates) and diverse representative alphasatellite isolates. The sequences obtained in this study are in red while those in black were taken from GenBank. Bootstrap analysis was performed with 1000 replicates. The matrix uses a discontinuous range of color (red, yellow, green, and blue) to differentiate two cut-off values representing the species (< 88% blue-green), and the isolate (> 88%, red) demarcation thresholds of alphasatellites.

Nine complete DNA satellite sequences (1215-1363 nt) and 1 partial sequence (1089-1148 nt), share nucleotide similarities of 75.91% to 87.87% with the alphasatellites available in GenBank. According to the species demarcation threshold of alphasatellites (88% nucleotide similarity) proposed Briddon et al. (2018), they are isolates of a new species (**Figure 6**) for which we propose the name ‘‘Asystasia yellow mosaic alphasatellite (AYMA)’’. These satellite components have typical features of alphasatellites, with a single replication gene in the virion sense, an A-rich region, and a stem-loop structure containing a non-nucleotide, TAGTATT↓AC. Pairwise comparisons using the SDT program showed that the alphasatellites genome sequences obtained in this work were closely related and showed 89.1–100.0% similarity.

A phylogenetic tree of alphasatellites was constructed using isolates obtained in this study and other representative alphasatellite sequences described elsewhere. However, phylogenetic analysis of the alphasatellite sequences is divided into two distinct clusters (**Figure 6**). The isolates of the first group were close to isolate MK032297 (Burkina Faso) and shared a nucleotide similarity of 87.87%; thus supporting our conclusion that these are a new species of alphasatellites. As for the second cluster, the isolates belonging to this group were closer to isolates MN614472 (Cameroon) and MK032294 (Burkina Faso) with about 75% nucleotide identity. The isolates of this study belonging to this second group were thus identified as new species of alphasatellites (**Figure 6**).

## 4 Discussion

Surveys were carried out in cassava fields in Côte d’Ivoire to determine which weeds or intercrops could be alternative host plants for Cassava mosaic begomoviruses (CMBs). Thus, plants showing typical CMD symptoms (mosaic, deformation, leaf curling, and filiform) were collected and analyzed by PCR or RCA and sequencing. The results showed that *A. gangetica* (Acanthaceae), *C. pubescens* (Fabaceae), *C. annuum* (Solanaceae), and *S. melongena* (Solanaceae) of three plant families are alternative hosts for CMBs. *A. gangetica* and *C. pubescens* were identified in almost all the agro-ecological zones of Côte d’Ivoire except for agro-ecological zones VI and VII located in the North. The two plant species were invasive in the cassava fields and showed mosaic symptoms like CMD. Indeed, *A. gangetica* is an invasive weed because of its capacity to produce large quantities of seeds, estimated at 27 million seeds per hectare (Priwiratama, 2011). *A. gangetica* is native to tropical Africa and Asia but has been introduced to many other tropical regions (Adetula, 2019). As for *C. pubescens*, a weed of cassava crops that can smother plants, it is a leguminous, herbaceous, creeping, and highly invasive plant that is often used as a cover crop to contribute to soil fertility restoration (Akedrin et al., 2010). *C. annuum* (Solanaceae) and *S. melongena* (Solanaceae) are food crops often grown in association with cassava. In the case of this study, those plants were found in 5 agro-ecological zones of Côte d’Ivoire (I, II, IV, V and VI).

This study revealed that two CMBs, *African cassava mosaic virus* (ACMV) and *East African cassava mosaic Cameroon virus* (EACMCMV) infect *A. gangetica*, *C. annuum*, and *S. melongena*. In *C. pubescens*, only ACMV was detected. Although these results confirmed that CMBs can infect other plants apart from cassava (Alabi et al., 2008 **;** Monde et al., 2010 **;** Eni et al., 2021). It is the first report from Côte d’Ivoire highlighting CMBs in *A. gangetica*, *C. pubescens*, *C. annuum*, and *S. melongena.* Peppers and other intercropping plants could also be hosts of CMBs. Our results are similar to those of (Dansou-Kodjo et al., 2019), those who showed that CMBs can infect crops other than cassava. As for the infection of CMBs in weeds, previous studies carried out in the Democratic Republic of Congo (Monde et al., 2010) and Nigeria (Ogbe et al., 2006 **;** Alabi et al., 2008 **;** Eni et al., 2021) revealed the presence of ACMV and EACMV in single and co-infection in weeds such as *C. pubescens, Chromolaena odorata* L., *Senna alata* (L.) Roxb., *S. occidentalis* (L.), *L. leucocephala* (Lam.), and many other weeds. Indeed, with high environmental adaptability, weeds are widely distributed worldwide and serve as reservoirs or alternative hosts for begomovirus survival, maintenance, and spread in the absence of major crops.

An important finding of this study is also a potential differential host-pathogen affinity between ACMV and EACMCMV and the hosts tested. Although ACMV infection was detected in all the hosts tested, it was not the case for EACMCMV which was not detected in *C. pubescens* at all and which seems to require ACMV to infect *A. gangetica* and *S. melongena.* Our results are similar to previous studies conducted across Central Africa (Monde et al., 2010) and West Africa (Eni and Fasasi, 2013) where *C. pubescens* was infected by ACMV. In addition, Leke et al. (2016) showed that ACMV infects *A. gangetica* in Nigeria. The current study suggests that EACMCMV may exhibit a better affinity to *C. annuum* L. compared to ACMV. Although the number of *C. annuum* L. samples tested was relatively low, it is clear that EACMCMV can readily infect this host without ACMV. The high rate of EACMCMV in *C. annuum* is surprising and could be explained by the emerging status of the virus formerly known to occur in mixed infection with ACMV that had evolved in a single infection (Kouakou et al., 2024). In East Africa, studies have identified wild hosts for cassava viruses not only among close relatives of cassava but also among unrelated (non-cassava) plant species. It is the case of *Manihot carthaginensis* subsp. *Glaziovii*, a wild cassava species, *Zanha Africana* (Radlk.) Exell and *Trichoderma zeylanicum* (Burm.f.) are unrelated (non-cassava) plant species that were found to be infected by Cassava brown streak virus (CBSV) and Ugandan Cassava Brown Streak Virus (UCBSV) in Mozambique (Amisse et al., 2019). Likewise, in rice, wild species such as *Oryza longstaminate* and *Oryza barthii* constitute natural reservoirs for the virus, facilitating the persistence of Rice yellow mottle virus (RYMV) in the environment between growing seasons (Bakonyi et al., 2012 **).** Tugume et al. (2010) also found wild species of genera *Ipomoea* to be infected by Sweet potato feathery mottle virus (SPFMV). These findings highlight the complexity of viral ecology and the critical role of uncultivated reservoirs in the spread of viruses.

In addition, our findings revealed the presence of non-CMBs and DNA satellites in weeds. Soybean chlorotic blotch virus (SbCBV) was detected in *C. pubescens*. This virus which naturally infects Soybeans is now found in *C. pubescens* as previously shown Alabi et al. (2010) in Nigeria. Several other non-CMBs that naturally infect crop plants have also been described in different weed species. These include *Tobacco rattle virus* (TRV) infecting *Chenopodium album, Amaranthus retroflexus, Solanum nigrum* L., *Sorghum halepense* L. (Dikova, 2006), *Cucumber mosaic virus* affecting cucurbit crops as well as wild cucurbits (*Cucumis* spp.) (Goyal et al., 2012), *Squash vein yellowing virus* infecting naturally squash and watermelon and also wild cucurbit weedy species like *Momordica charantia* (Shrestha et al., 2016). Furthermore, in this work, *A. gangetica* was infected by West African Asystasia virus 1 (WAAV1; bipartite begomovirus), West African Asystasia virus 2 (WAAV2; monopartite begomovirus) and several alphasatellites which have been classified as new species with the proposed name ‘‘Asystasia yellow mosaic alphasatellite (AYMA)’’. Cases of co-infection of CMBs with WAAV1 and AYMA were observed. Co-infections between several viruses in the same plant species are likely to create new viral species, through recombination, reassortment, or other phenomena (Rocha et al., 2013 **;** Saleem et al., 2016 **;** Lima et al., 2017). *A. gangetica* therefore appears to be the plant species that could favor an emergence of new viral species damaging to cassava cultivation in regions where the plant is widespread.

The occurrence of WAAV1 and WAAV2 in *A. gangetica* has already been reported in a study by Wyant et al. (2015). In Madagascar, another begomovirus has been identified infecting *A. gangetica*, with a bipartite genome organization, named Asystasia mosaic Madagascar virus (AMMGV), previous studies reported the existence of a new begomovirus species named West African Asystasia virus 3 in *A. gangetica* in Benin (De Bruyn et al., 2015 **;** Lauryn et al., 2023). According to these authors, *A. gangetica* is a major host of begomovirus complexes in West and Central Africa. Also, the identification of alphasatellites in weeds has been reported in West and Central Africa (Leke et al., 2015). In addition, studies by Harimalala et al. (2013) and Leke et al. (2015) reported the presence of alphasatellites, SbCBV and WAAV1 in cassava. This indicates that alphasatellites and these begomoviruses can infect cassava. These results may be due to the genomic plasticity of begomoviruses, which enables them to adapt to new environments and hosts (Navas-Castillo et al., 2011), and to the presence of polyphagous populations of *B. tabaci*, the vector of these viruses. Previous studies have shown that *B. tabaci* exhibits a flight behavior that promotes migration between different plant species, including cassava and weeds (Chikoti et al., 2019). The duration during which *B. tabaci* can carry and transmit a begomovirus varies among virus strains and host species (Navas-Castillo et al., 2011). Thus, cassava’s proximity to weeds and intercropping exposes it to multiple viral infections. Off the cassava cultivation season, the weeds and intercrops described here host cassava-infecting viruses as a reservoir, which re-infect cassava during cassava production seasons. This first study in Côte d’Ivoire will constitute a basis for informed recommendations and good practices on weed management in and around cassava fields, and the need to carefully choose the plants to intercrop with cassava to enhance disease management. Control of alternative hosts of begomoviruses responsible for CMD requires an integrated approach to limit the spread of the viruses. Weeds acting as reservoirs should be eliminated regularly. Awareness campaigns among farmers are necessary to identify alternative hosts of begomoviruses and adopt appropriate management measures.

## 5 Conclusion

This work highlights *A. gangetica*, *C. pubescens*, *C. annuum*, and *S. melongena* as alternative hosts of begomoviruses which contribute to the maintenance of CMD in Côte d’Ivoire. Also, *A. gangetica* carries other begomoviruses constituting a threat to cassava cultivation. It is important to train farmers about the role of these alternative hosts and advise them to regularly plow their cassava plots to prevent the spread of the disease.

## Data Availability

The datasets presented in this study can be found in online repositories. The names of the repository/repositories and accession number(s) can be found in the article/supplementary material.
